# Oral Health of Patients With Inflammatory Bowel Disease—A Cross‐Sectional Survey in Austria

**DOI:** 10.1002/cre2.70398

**Published:** 2026-08-02

**Authors:** Kristina Bertl, Arghavan Yari, Anna Riener, Johan Burisch, Nikolaos Pandis, Hady Haririan, Andreas Stavropoulos

**Affiliations:** ^1^ Department of Periodontology, Faculty of Medicine, Dental Clinic Sigmund Freud University Vienna Vienna Austria; ^2^ Department of Periodontology Blekinge Hospital Karlskrona Sweden; ^3^ Dental Education Unit, University Clinic of Dentistry Medical University of Vienna Vienna Austria; ^4^ Gastrounit, Medical Division Copenhagen University Hospital ‐ Amager and Hvidovre Hvidovre Denmark; ^5^ Copenhagen Center for Inflammatory Bowel Disease in Children, Adolescents and Adults Copenhagen University Hospital ‐ Amager and Hvidovre Hvidovre Denmark; ^6^ Faculty of Health and Medical Sciences, Clinical Medicine University of Copenhagen Copenhagen Denmark; ^7^ Department of Orthodontics and Dentofacial Orthopedics, School of Dental Medicine University of Bern Bern Switzerland; ^8^ Periodontology, Faculty of Odontology University of Malmö Malmö Sweden; ^9^ Division of Conservative Dentistry and Periodontology, University Clinic of Dentistry Medical University of Vienna Vienna Austria; ^10^ Department of Periodontology, School of Dental Medicine University of Bern Bern Switzerland

**Keywords:** Crohn's disease, inflammatory bowel disease, oral health, survey, ulcerative colitis

## Abstract

**Objective:**

This cross‐sectional survey aimed to assess oral health in patients with inflammatory bowel diseases (IBD) living in Austria.

**Methods:**

Data collection was conducted with an online questionnaire, including when possible validated tools, covering general medical history, socioeconomic factors, IBD‐related parameters, and various oral health parameters, that is, tooth loss rate, prevalence of severe periodontitis, oral health‐related quality of life (OHRQoL), and presence of oral lesions. The questionnaire was disseminated to the members of the Austrian Crohn's Disease/Ulcerative Colitis Association.

**Results:**

The analysis included the responses of 327 patients (68% female; average age 46 years), 134 with ulcerative colitis (UC) or IBD unclassified, and 193 with Crohn's disease (CD). Based on self‐reported data, about 85% of the patients reported intake of an IBD‐related medication and about two thirds of the responders reported some IBD activity in the past 12 months. Approximately 24% of the patients had < 20 remaining teeth, 34% had severe periodontitis, and 7% reported poor OHRQoL. IBD diagnosis did not affect the odds for having < 20 teeth or self‐reported severe periodontitis, but patients with CD presented more frequently with poor OHRQoL. In addition, patients with CD received more frequently information from their physician on the possibility of oral lesions due to IBD; however, > 50% of all patients indicated not having any problems with oral lesions.

**Conclusions:**

Oral health remains inadequately addressed in patients with IBD despite the significant need for dental care. Hence, treating physicians should motivate patients with IBD to inform their dentist about their diagnosis and to maintain regular dental checkups.

## 1 Introduction

There has been a growing interest in exploring the link between inflammatory bowel diseases (IBD), such as ulcerative colitis (UC) and Crohn's disease (CD), and oral diseases, such as periodontal diseases. This is reflected in numerous preclinical and clinical studies as well as numerous (systematic) reviews on this topic (Agossa et al. 2017; She et al. 2020; Lorenzo‐Pouso et al. 2021; Zhang et al. 2021; Nijakowski et al. 2021; Agossa et al. 2021; Domokos et al. 2022; Akash et al. 2025). Overall, it appears that patients with IBD have an increased risk of developing periodontitis and/or have more periodontal problems, while yet limited evidence suggests that periodontitis may also increase the risk for IBD (Lin et al. 2018; Kang et al. 2020). Both diseases are chronic inflammatory diseases in a susceptible host initiated by a bacterial trigger and leading to destruction of the surrounding tissues (Graves 2008; Cho 2008; Indriolo et al. 2011; Bartold and Van Dyke 2013; Bertl et al. 2016; de Souza and Fiocchi 2016); however, the exact mechanisms behind this association are still discussed. One proposed mechanism is that periodontitis‐associated bacteria translocate from the oral cavity to the colon and aggravate the inflammatory process. This theory has been proven experimentally in preclinical models, mostly for *Fusobacterium nucleatum* and partly also for other oral bacteria (e.g., *Porphyromonas gingivalis*) (Akash et al. 2025).

As the scientific knowledge regarding the association between IBD and periodontitis has historically been based mostly on small and heterogeneous cohorts, our group initiated recently a large‐scale, questionnaire‐based case‐control study, initially conducted in Denmark. This survey was completed by approximately 1100 patients with IBD and 3400 individuals without IBD (Bertl, Burisch et al. 2022; Madsen et al. 2023; Bertl, Burisch et al. 2024a; Bertl, Tsakos et al. 2023). This study demonstrated that patients with IBD had significantly poorer oral health, including a higher prevalence of self‐reported severe periodontitis and tooth loss rate, compared to non‐IBD individuals (Bertl et al. 2022). The poorer oral health of patients with IBD was also associated with more frequent dental visits and higher costs related to dental treatments (Bertl et al.2024a). Importantly, patients with IBD and periodontitis experienced higher IBD disease activity and greater IBD‐related disability compared to periodontally healthy IBD patients (Madsen et al. 2023). Subsequently, a similar survey was run in Sweden, including almost 800 patients with IBD, and the findings from the Danish population were consistently replicated (Bertl, Burisch et al. 2024b; Madsen et al. 2025).

In this context, the prevalence of IBD in Scandinavia is among the highest in the world, while dental care is, in general, of high level, something reflected in the relatively high level of periodontal health (König et al. 2010); for instance, in Sweden patients with IBD receive an annual special dental care allowance. It is thus of interest to collect information on oral health of patients with IBD from a central European country with a lower prevalence of IBD and a different dental care system than Scandinavia. Hence, the objective of the present cross‐sectional study was to extend our knowledge on oral health in patients with IBD in a central European country, utilizing the same online questionnaire that was previously used in Denmark and Sweden.

## 2 Materials and Methods

### 2.1 Distribution of the Questionnaire

This cross‐sectional survey was performed among patients with IBD living in Austria, and reporting complies with the STROBE guidelines. The questionnaire mirrored the survey previously distributed in Denmark and Sweden (Bertl et al. 2022; Madsen et al. 2023; Bertl et al. 2024a; Bertl et al. 2023; Bertl et al. 2024b; Madsen et al. 2025) and included different aspects, that is, general, IBD‐related, and oral health‐related questions. It was either forward‐translated into German or the available German version of the various tools was used. It was disseminated via mail, social media, and an announcement on the homepage to the members of the ÖMCCV (Austrian Crohn's Disease/Ulcerative Colitis Association) (https://www.oemccv.at/). Anonymous data collection took place over a 5‐month period, from June to October 2022, using a web‐based platform (Sunet Survey) and 2 reminders were sent during this period; ethical approval was not required by the local authorities.

### 2.2 Content of the Questionnaire—General Information

The survey included questions on gender, age, body height and weight (to calculate body mass index [BMI]), smoking status (i.e., never, current, or former smoker), and the presence/absence of various comorbidities. In addition, the questionnaire covered details about the living area, educational background, and approximate income per month (after taxes).

### 2.3 Content of the Questionnaire—IBD‐Related Information

The following information on patients' IBD was collected: IBD diagnosis (CD, UC, or IBD unclassified [IBD‐U]), years since diagnosis, intake of any IBD medication and type of medication, any previous surgery due to IBD, and the presence of a stoma. Patients with CD were also asked about disease localization (small/large intestines, terminal ileum, or combinations thereof). Further, disease activity in the past 12 months was recorded as well as validated questionnaires for disability, disease activity (separate for UC and CD), and quality of life. Specifically, disability was assessed by the IBD Disability Index (IBD‐DI) (Lo, Julsgaard et al. 2018a, 2018b; Lo, Prosberg et al. 2018b), categorized as none (≤ 13), mild (14–26), moderate (27–42), and severe (≥ 43). Disease activity was evaluated by the Simple Clinical Colitis Activity Index (SCCAI) (Walmsley et al. 1998) for UC and the Harvey‐Bradshaw Index (HBI) (Harvey and Bradshaw 1980) for CD, with standard cut‐offs applied (SCCAI: remission 0–2, mild 3–5, moderate 6–11, severe ≥ 12; HBI: inactive ≤ 5, active 6–7, very active ≥ 8). Finally, disease‐related quality of life was measured using the short Inflammatory Bowel Disease Questionnaire (s‐IBDQ) (Irvine et al. 1996; Jowett et al. 2001; Christiansen et al. 2019).

### 2.4 Content of the Questionnaire—Oral Health‐Related Information

For oral health‐related characteristics, the following information was gathered: number of remaining teeth, self‐perceived state of teeth and gums, presence of any tooth replacement, details on regular oral hygiene measures, reasons for and time since the last dental check‐up, and money spent on professional dental care in the past 12 months. Further, questions for self‐reported measures for periodontitis surveillance (SRMSP) (Eke et al. 2013), oral health‐related quality of life (OHRQoL), and the presence of self‐reported severe periodontitis were included. Some of the OHRQoL questions were derived from the World Health Organization (2013), and also the 5 questions from the Oral Health Impact Profile (OHIP‐5) were used (John et al. 2006; Wide and Hakeberg 2018). For OHIP, the total sum (i.e., 0 to 20) was calculated, and OHRQoL was judged dichotomously as good or poor, that is, if more than one oral impact was experienced “fairly often” or “very often,” the responder was judged as having a poor OHRQoL (Wide and Hakeberg 2018). The presence/absence of self‐reported severe periodontitis was judged with the Periodontal Screening Score (PESS) (Carra et al. 2018). This score is based on 5 questions for self‐reported surveillance of periodontitis, age, and smoking status, and a score ≥ 5 is indicative for severe periodontitis (Carra et al. 2018). Finally, details on the patients' experience with IBD‐related oral lesions were collected.

### 2.5 Statistical Analysis

For statistical analysis, patients with IBD‐U were included in the group of patients with UC. Data were reported separately for patients with UC and CD, and distribution of data was assessed graphically using Q‐Q‐plots and the Shapiro–Wilk test. Categorical variables were expressed as frequencies and continuous variables as mean (standard deviation) or median (first and third quartile). Fisher's exact test or chi‐squared test was used for categorical parameters, and either the independent *t*‐test or the Mann–Whitney *U* test for continuous variables to assess any differences between UC and CD.

The following 2 primary dichotomous outcome parameters were defined and assessed with binary logistic regression models: (1) PESS (score < 5 vs. ≥ 5), and (2) tooth number (≥ 20 teeth vs. < 20 teeth). Patient group (UC/CD) was defined as the main predictor and age, gender, and smoking status as à priori confounders. In addition, the following confounders were considered in the models: (1) diabetes (yes/no); (2) osteoporosis (yes/no); (3) rheumatoid arthritis (yes/no); (4) ankylosing spondylitis (yes/no); (5) psoriasis (yes/no); (6) depression (yes/no); (7) high cholesterol (yes/no); (8) cardiovascular disease (yes/no); (9) asthma (yes/no); (10) chronic obstructive pulmonary disease (COPD; yes/no); (11) living area (Vienna/> 100,000 inhabitants/40,000 to 100,000 inhabitants/10,000 to 40,000 inhabitants/< 10,000 inhabitants); (12) education (no school or primary school/high school/higher education up to 3 years/higher education up to 6 years and/or PhD); (13) income after taxes (< 1000 €/1000 to < 2000 €/2000 to < 3000 €/ ≥ 3000 €); and (14) BMI. In the analysis for PESS, “tooth number” (≥ 20 teeth/< 20 teeth) and, in the analysis for tooth number, “PESS” (score < 5/ ≥ 5) was added as additional confounder. Separate univariable analyses, corrected for the à priori confounders, were run for the main predictor and each confounder. Multivariable analyses were performed for the main predictor, the à priori confounders, and all confounders presenting a *p*‐value < 0.2 in the univariable analysis. Odds ratio (OR) and 95% confidence interval (CI) were calculated, and the fit of the models in the final multivariable analyses was confirmed with the Hosmer–Lemeshow test (i.e., *p* > 0.05). Statistical analysis was performed with STATA/IC 17.0 for Mac, and a *p*‐value of ≤ 0.05 was considered statistically significant.

## 3 Results

### 3.1 Study Population and General Characteristics

In total, 327 patients with IBD answered the questionnaire; 193 with CD, 132 with UC, and 2 with IBD‐U. The general characteristics of these patients are summarized in Table 1. Briefly, most participants were female, with a significantly higher proportion among patients with CD compared to patients with UC. The median age was about 44 years, and the most prevalent comorbidity was depression. Comorbidities presented overall a similar prevalence among patients with CD and UC, except for psoriasis, which was significantly more prevalent among patients with CD. About half of the responders were former smokers; never smokers were significantly more and current smokers significantly less prevalent among UC patients. About one third of the responders were living in the capital (Vienna), and about one third had finished a higher education; patients with UC presented with a significantly higher income.

**Table 1:** Self‐reported general characteristics of patients with ulcerative colitis (including unclassified inflammatory bowel disease; *n* = 134) and Crohn's disease (*n* = 193).

Parameter		Ulcerative colitis	Crohn's disease	*p* value^a^
Gender (*n* [%])	Female	78 (58.2)	143 (74.1)	**< 0.01**
	Male	56 (41.8)	50 (25.9)	
Age	Median (Q1; Q3)	43 (34; 57)	47 (36; 56)	0.50
BMI	Median (Q1; Q3)	24.8 (21.9; 27.3)	23.8 (21.1; 27.2)	0.16
Comorbidities (yes; *n* [%])	Diabetes	8 (6.0)	3 (1.6)	0.06^b^
	Osteoporosis	8 (6.0)	17 (8.8)	0.34
	Rheumatoid arthritis	6 (4.5)	11 (5.7)	0.63
	Ankylosing spondylitis	2 (1.5)	4 (2.1)	1.00^b^
	Psoriasis	3 (2.2)	15 (7.8)	**0.05** b
	Depression	16 (11.9)	32 (16.6)	0.24
	High cholesterol	15 (11.2)	19 (9.8)	0.69
	Cardiovascular disease	7 (5.2)	7 (3.6)	0.48
	Asthma	7 (5.2)	8 (4.2)	0.65
	COPD	2 (1.5)	3 (1.6)	1.00^b^
Smoking (*n* [%])	Never	60 (44.8)	61 (31.6)	**< 0.01**
	Former	63 (47.0)	89 (46.1)	
	Current	11 (8.2)	43 (22.3)	
Living area (*n* [%])	Vienna	35 (26.1)	63 (32.6)	0.62^b^
	> 100,000 inhabitants	13 (9.7)	18 (9.3)	
	40,000 to 100,000 inhabitants	4 (3.0)	8 (4.2)	
	10,000 to 40,000 inhabitants	19 (14.2)	29 (15.0)	
	< 10,000 inhabitants	63 (47.0)	75 (38.9)	
Education (*n* [%])	No school	1 (0.8)	0 (0.0)	0.09^b^
	Primary school	1 (0.8)	10 (5.2)	
	Vocational training	31 (23.1)	56 (29.0)	
	High school	49 (36.5)	71 (36.8)	
	Higher education up to 3 years	22 (16.4)	19 (9.8)	
	Higher education up to 6 years	25 (18.7)	32 (16.6)	
	PhD	5 (3.7)	5 (2.6)	
Income per month (*n* [%])	**<** 1000 €	22 (16.4)	28 (14.5)	**0.02**
	1000 to < 2000 €	44 (32.8)	96 (49.7)	
	2000 to < 3000 €	47 (35.1)	52 (27.0)	
	≥ 3000 €	21 (15.7)	17 (8.8)	

*Note:* Bold values indicate significance.

Abbreviations: €, euros; BMI, body mass index; COPD, chronic obstructive pulmonary disease; Q1/Q3, first/third quartile; SD, standard deviation.

^a^ Chi‐squared test for categorical variables and Mann–Whitney *U* test for continuous variables were applied unless indicated otherwise.

^b^ Fisher's exact test was applied.

### 3.2 IBD‐Related Characteristics

Table 2 summarizes the IBD‐related characteristics of the responders. The average time since IBD diagnosis ranged between 14 and 18 years and was significantly longer for patients with CD. About 85% of the responders were currently taking a medication due to IBD; about 60% of the patients with UC were taking 5‐aminosalicylic acids, while about 67% of the patients with CD were prescribed some type of biological therapy. In addition, patients with CD had a significantly higher rate of previous surgery due to IBD. About two‐thirds of the responders reported some disease activity in the past 12 months, mostly once or twice, and about one‐third each was classified as experiencing either a moderate or severe degree of disability. Nevertheless, about 40% of the patients with CD and UC were classified as in remission/being inactive. Finally, disease‐related quality of life was significantly more negatively affected for patients with CD regarding social functioning and systemic symptoms.

**Table 2:** Self‐reported IBD‐related characteristics of patients with ulcerative colitis (including unclassified inflammatory bowel disease; *n* = 134) and Crohn's disease (*n* = 193).

Parameter		Ulcerative colitis	Crohn's disease	*p* value^a^
Years since diagnosis of IBD	Median (Q1; Q3)	10 (5; 22)	18 (7; 29)	**< 0.01**
Intake of medication for treatment of IBD (*n* [%])	Yes	114 (85.1)	166 (86.0)	0.81
	No	20 (14.9)	27 (14.0)	
Current IBD medication (*n* [%])^c^	5‐aminosalicylic acid	81 (60.5)	38 (19.7)	**< 0.01**
	Prednisolone	11 (8.2)	7 (3.6)	0.07
	Topical drugs	4 (3.0)	9 (4.7)	0.57^b^
	Immunotherapy	13 (9.7)	18 (9.3)	0.91
	Biological therapy	57 (42.5)	130 (67.4)	**< 0.01**
Previous surgery due to IBD (*n* [%])	Yes	17 (12.7)	111 (57.5)	**< 0.01**
	No	117 (87.3)	82 (42.5)	
Presence of a stoma (*n* [%])	Yes	3 (2.2)	14 (7.3)	0.12^b^
	Yes (but not anymore)	10 (7.5)	12 (6.2)	
	No	121 (90.3)	167 (86.5)	
Episodes of disease activity in the last 12 months (*n* [%])	None	42 (31.3)	54 (28.0)	0.30
	Once	17 (12.7)	39 (20.2)	
	Twice	28 (20.9)	28 (14.5)	
	3‐times	8 (6.0)	21 (10.9)	
	4‐times	7 (5.2)	10 (5.2)	
	5‐times	7 (5.2)	8 (4.1)	
	6‐times or more	25 (18.7)	33 (17.1)	
Localization of Crohn's disease (*n* [%])	Small intestine		21 (10.9)	—
	Terminal ileum		87 (45.1)	
	Large intestine		26 (13.5)	
	Small and large intestine		52 (26.9)	
	Don't know		7 (3.6)	
IBD disability index score	Median (Q1; Q3)	32.1 (17.9; 48.2)	35.7 (25.0; 50.0)	0.06
IBD disability index (*n* [%])	None	15 (11.2)	12 (6.2)	0.22
	Mild	35 (26.1)	41 (21.2)	
	Moderate	40 (29.9)	64 (33.2)	
	Severe	44 (32.8)	76 (39.4)	
SCCAI categories (*n* [%])	Remission	57 (42.6)		—
	Mild	42 (31.3)		
	Moderate	33 (24.6)		
	Severe	2 (1.5)		
Harvey and Bradshaw's Activity index (*n* [%])	Inactive		85 (44.0)	—
	Active		25 (13.0)	
	Very active		83 (43.0)	
sIBDQ (median [Q1; Q3])	Bowel symptoms	15 (11; 18)	14 (12; 18)	0.72
	Emotional functioning	14 (12; 16)	13 (12; 15)	0.21
	Social functioning	12 (9; 14)	11 (8; 13)	**0.04**
	Systemic symptoms	9 (7; 10)	8 (6; 10)	**0.02**
	Total score	48 (42; 57)	46 (39; 53)	0.11

*Note:* Bold values indicate significance.

Abbreviations: IBD, inflammatory bowel disease; Q1/Q3, first/third quartile; SCCAI, Simple Clinical Colitis Activity Index; SD, standard deviation; sIBDQ, short Inflammatory Bowel Disease Questionnaire.

^a^ Chi‐squared test for categorical variables and Mann–Whitney *U* test for continuous variables were applied unless indicated otherwise.

^b^ Fisher's exact test was applied.

^c^ Some responders received more than one type of IBD medication.

### 3.3 Dental and Periodontal Characteristics

Table 3 summarizes the self‐reported dental and periodontal characteristics and dental care habits. About 1 out of 4 responders reported having < 20 teeth left, and about one third reported suffering from severe periodontitis. Significantly more patients with CD judged the overall health of their teeth and gums as average or bad, and they also spent significantly more money on professional dental care in the past 12 months compared to patients with UC. About one fourth of the responders had dental implants; oral hygiene measures were well comparable between patients with CD and UC, and 4 out of 5 patients had been visiting a dentist in the past 12 months, mostly for a routine check‐up.

**Table 3:** Self‐reported dental and periodontal characteristics and dental care habits of patients with ulcerative colitis (including unclassified inflammatory bowel disease; *n* = 134) and Crohn's disease (*n* = 193).

Parameter		Ulcerative colitis	Crohn's disease	*p* value^a^
Tooth number (*n* [%])	Edentulous	3 (2.2)	4 (2.1)	0.54^b^
	1 to 9 teeth	7 (5.2)	18 (9.3)	
	10 to 19 teeth	18 (13.4)	29 (15.0)	
	≥ 20 teeth	106 (79.2)	142 (73.6)	
PESS ≥ 5 (yes; *n* [%])	All patients	42 (31.3)	68 (35.2)	0.46
	< 40 years	2 (3.8)	5 (7.0)	—
	40 to 54 years	14 (31.8)	20 (29.9)	
	≥ 55 years	26 (70.3)	43 (78.2)	
PESS (*n* [%])	1 to 4	92 (68.7)	125 (64.8)	**< 0.01** b
	5 to 8	40 (29.8)	46 (23.8)	
	9 to 13	2 (1.5)	22 (11.4)	
Overall, how would you rate the health of your teeth and gums? (*n* [%])	Excellent	6 (4.5)	2 (1.0)	**< 0.01** b
	Very good	32 (23.9)	26 (13.5)	
	Good	45 (33.6)	50 (25.9)	
	Average	27 (20.1)	77 (39.9)	
	Bad	24 (17.9)	38 (19.7)	
	Do not know	0 (0.0)	0 (0.0)	
Tooth replacement (present; *n* [%])^c^	Partial denture	12 (9.0)	24 (12.4)	0.32
	Full upper denture	7 (5.2)	7 (3.6)	0.48
	Full lower denture	6 (4.5)	4 (2.1)	0.33^b^
	Dental implants	34 (25.4)	49 (25.4)	1.00
Frequency of toothbrushing (*n* [%])	Never	0 (0.0)	1 (0.5)	0.55^b^
	Once a month	1 (0.8)	0 (0.0)	
	2‐ to 3‐times a month	0 (0.0)	0 (0.0)	
	Once a week	0 (0.0)	0 (0.0)	
	2‐ to 6‐times a week	0 (0.0)	2 (1.0)	
	Once a day	38 (28.3)	59 (30.6)	
	Twice or more a day	95 (70.9)	131 (67.9)	
Oral hygiene measures (used; *n* [%])^c^	Wooden toothpick	38 (28.4)	63 (32.6)	0.41
	Plastic toothpick	12 (9.0)	26 (13.5)	0.21
	Thread (dental floss)	84 (62.7)	116 (60.1)	0.64
	Interdental brush	50 (37.3)	77 (39.9)	0.64
	Charcoal	2 (1.4)	9 (4.6)	0.21^b^
	Chewstick/Miswak	0 (0.0)	0 (0.0)	—
	Toothpaste	132 (98.5)	192 (99.5)	0.57^b^
Last dental visit (*n* [%])	≤ 6 months	82 (61.2)	112 (58.0)	0.08^b^
	6 to 12 months ago	27 (20.2)	46 (23.8)	
	1 to 2 years ago	19 (14.2)	14 (7.3)	
	2 to 5 years ago	5 (3.7)	17 (8.8)	
	≥ 5 years ago	1 (0.7)	4 (2.1)	
	Never	0 (0.0)	0 (0.0)	
Reason for last dental visit (*n* [%])	Consultation	1 (0.8)	2 (1.0)	0.06^b^
	Pain and/or oral health problems	33 (24.6)	64 (33.2)	
	Treatment	25 (18.6)	47 (24.3)	
	Regular check‐up	75 (56.0)	80 (41.5)	
	Don't know	0 (0.0)	0 (0.0)	
Money spent for professional dental care in the last 12 months (*n* [%])^d^	< 500 €	81 (74.3)	102 (64.5)	**0.03** b
	500 to < 1000 €	13 (11.9)	33 (20.9)	
	1000 to < 3000 €	13 (11.9)	11 (7.0)	
	3000 to < 6000 €	2 (1.9)	7 (4.4)	
	6000 to < 10,000 €	0 (0.0)	5 (3.2)	
	≥ 10,000 €	0 (0.0)	0 (0.0)	

*Note:* Bold values indicate significance.

Abbreviations: €, euros; PESS, periodontal screening score.

^a^ Chi‐squared test was applied unless indicated otherwise.

^b^ Fisher's exact test was applied.

^c^ Multiple answers possible.

^d^ Based on 267 responders, who have been with the dentist in the last 12 months.

### 3.4 Oral‐Health Related QoL Aspects

The oral‐health related QoL aspects are summarized in Table 4. Overall, there were no significant differences for the WHO‐derived questions between patients with CD and UC and most of the questions were answered by the responders with either “no” or “sometimes.” However, the OHIP sum indicated a significantly more severely affected OHRQoL in patients with CD, and a poor OHRQoL was 3‐times more frequent in patients with CD compared to patients with UC. Further, among the OHIP‐5 questions, about every fourth to fifth responder reported having occasional difficulties chewing any food due to problems with their teeth, mouth, dentures or jaws and having occasional pain in their mouth. In addition, patients with CD reported significantly more frequently feeling uncomfortable about the appearance of their teeth or dentures.

**Table 4:** Self‐reported oral‐health related quality of life aspects of patients with ulcerative colitis (including unclassified inflammatory bowel disease; *n* = 134) and Crohn's disease (*n* = 193).

Parameter			Ulcerative colitis	Crohn's disease	*p* value^a^
Because of the state of your teeth or mouth, how often have you experienced any of the following problems during the past 12 months?^c^	Difficulty in biting foods (*n* [%])	Very often	2 (1.5)	10 (5.2)	0.20
		Fairly often	4 (3.0)	12 (6.2)	
		Sometimes	35 (26.1)	53 (27.5)	
		No	92 (68.6)	115 (59.6)	
		Don't know	1 (0.8)	3 (1.5)	
	Difficulty chewing foods (*n* [%])	Very often	2 (1.5)	6 (3.1)	0.15
		Fairly often	6 (4.5)	9 (4.7)	
		Sometimes	29 (21.6)	61 (31.6)	
		No	96 (71.6)	113 (58.5)	
		Don't know	1 (0.8)	4 (2.1)	
	Difficulty with speech/trouble pronouncing words (*n* [%])	Very often	0 (0.0)	1 (0.5)	0.26
		Fairly often	4 (3.0)	3 (1.6)	
		Sometimes	8 (6.0)	23 (11.9)	
		No	119 (88.8)	160 (82.9)	
		Don't know	3 (2.2)	6 (3.1)	
	Dry mouth (*n* [%])	Very often	4 (3.0)	15 (7.8)	0.15
		Fairly often	8 (6.0)	21 (10.9)	
		Sometimes	49 (36.5)	63 (32.6)	
		No	71 (53.0)	89 (46.1)	
		Don't know	2 (1.5)	5 (2.6)	
	Felt embarrassed due to appearance of teeth (*n* [%])	Very often	7 (5.2)	12 (6.2)	0.06
		Fairly often	4 (3.0)	16 (8.3)	
		Sometimes	25 (18.7)	52 (26.9)	
		No	91 (67.9)	107 (55.5)	
		Don't know	7 (5.2)	6 (3.1)	
	Felt tense because of problems with teeth or mouth (*n* [%])	Very often	4 (3.0)	12 (6.2)	0.74
		Fairly often	9 (6.7)	13 (6.7)	
		Sometimes	42 (31.4)	63 (32.7)	
		No	74 (55.2)	99 (51.3)	
		Don't know	5 (3.7)	6 (3.1)	
	Have avoided smiling because of teeth (*n* [%])	Very often	2 (1.5)	12 (6.2)	0.08
		Fairly often	10 (7.5)	14 (7.3)	
		Sometimes	12 (8.9)	28 (14.5)	
		No	108 (80.6)	138 (71.5)	
		Don't know	2 (1.5)	1 (0.5)	
	Had sleep that is often interrupted (*n* [%])	Very often	11 (8.2)	26 (13.5)	0.56^b^
		Fairly often	17 (12.7)	22 (11.4)	
		Sometimes	33 (24.6)	52 (26.9)	
		No	68 (50.8)	88 (45.6)	
		Don't know	5 (3.7)	5 (2.6)	
	Have taken days off work (*n* [%])	Very often	0 (0.0)	5 (2.6)	0.21
		Fairly often	3 (2.2)	1 (0.5)	
		Sometimes	17 (12.7)	28 (14.5)	
		No	109 (81.4)	149 (77.2)	
		Don't know	5 (3.7)	10 (5.2)	
	Difficulty doing usual activities (*n* [%])	Very often	1 (0.8)	9 (4.7)	0.06
		Fairly often	6 (4.4)	8 (4.1)	
		Sometimes	24 (17.9)	46 (23.8)	
		No	102 (76.1)	124 (64.3)	
		Don't know	1 (0.8)	6 (3.1)	
	Felt less tolerant of spouse or people who are close to you (*n* [%])	Very often	1 (0.7)	4 (2.1)	0.36
		Fairly often	6 (4.5)	10 (5.2)	
		Sometimes	22 (16.4)	44 (22.8)	
		No	99 (73.9)	122 (53.2)	
		Don't know	6 (4.5)	13 (6.7)	
	Have reduced participation in social activities (*n* [%])	Very often	2 (1.5)	7 (3.6)	0.21
		Fairly often	12 (9.0)	15 (7.8)	
		Sometimes	22 (16.4)	46 (23.8)	
		No	95 (70.9)	124 (64.3)	
		Don't know	3 (2.2)	1 (0.5)	
Within the last 7 days…	**…**have you had difficulty chewing any food because of problems with your teeth, mouth, dentures or jaws? (*n* [%])	Very often	3 (2.2)	4 (2.1)	0.07
		Fairly often	4 (3.0)	11 (5.7)	
		Occasionally	26 (19.4)	51 (26.4)	
		Hardly ever	17 (12.7)	37 (19.2)	
		Never	84 (62.7)	90 (46.6)	
		Don't know	0 (0.0)	0 (0.0)	
	…have you had painful aching in your mouth? (*n* [%])	Very often	2 (1.5)	7 (3.6)	0.36
		Fairly often	7 (5.2)	18 (9.3)	
		Occasionally	34 (25.4)	49 (25.4)	
		Hardly ever	29 (21.6)	45 (23.3)	
		Never	61 (45.5)	74 (38.4)	
		Don't know	1 (0.8)	0 (0.0)	
	…have you felt uncomfortable about the appearance of your teeth or dentures? (*n* [%])	Very often	1 (0.8)	11 (5.7)	**0.02**
		Fairly often	7 (5.2)	9 (4.7)	
		Occasionally	12 (8.9)	35 (18.1)	
		Hardly ever	17 (12.7)	22 (11.4)	
		Never	96 (71.6)	115 (59.6)	
		Don't know	1 (0.8)	1 (0.5)	
	…have you felt that there has been less flavor in your food because of problems with your teeth, mouth, dentures or jaws? (*n* [%])	Very often	0 (0.0)	3 (1.6)	0.48
		Fairly often	2 (1.5)	6 (3.1)	
		Occasionally	7 (5.2)	14 (7.2)	
		Hardly ever	17 (12.7)	22 (11.4)	
		Never	106 (79.1)	141 (73.1)	
		Don't know	2 (1.5)	7 (3.6)	
	…have you had difficulty doing your usual jobs because of problems with your teeth, mouth, dentures or jaws? (*n* [%])	Very often	0 (0.0)	1 (0.5)	0.66
		Fairly often	0 (0.0)	3 (1.6)	
		Occasionally	2 (1.5)	5 (2.6)	
		Hardly ever	12 (8.9)	22 (11.4)	
		Never	119 (88.8)	160 (82.9)	
		Don't know	1 (0.8)	2 (1.0)	
OHIP judgment (*n* [%])		Poor	5 (3.7)	19 (9.8)	**0.04** b
OHIP sum		Median (Q1; Q3)	2 (0; 4)	2 (1; 6)	**< 0.01**

*Note:* Bold values indicate significance.

Abbreviations: OHIP, oral health impact profile; Q1/Q3, first/third quartile; SD, standard deviation.

^a^ Fisher's exact test for categorical variables and Mann–Whitney *U* test for continuous variables were applied unless indicated otherwise.

^b^ Chi‐squared test was applied.

^c^ Questions are derived from the World Health Organization (WHO) Oral Health Questionnaire.

### 3.5 Experience With Oral Lesions

Table 5 summarizes the responders' experience with oral lesions. Patients with CD were significantly more often informed by their physician about the connection between IBD and oral lesions; they tended to judge their physician as more likely to be interested in problems related to oral health, and they received significantly more frequently some kind of treatment for their oral lesions, mostly from their dentist. The prevalence of oral lesions did not differ significantly between patients with CD and UC. Less than 50% of the patients indicated having oral lesions, which were mostly localized (i.e., about 65%), slightly or average painful (i.e., about 70% to 81%), seldom or sometimes preventing them from eating (i.e., about 53% to 67%), and never or seldom negatively affecting social activities (i.e., about 69% to 77%). Further, the lesions were not related to IBD symptoms (i.e., about 60% to 63%), and no significant differences were noted between patients with CD and UC.

**Table 5:** Experience with oral lesions among patients with ulcerative colitis (including unclassified inflammatory bowel disease; *n* = 134) and Crohn's disease (*n* = 193).

Parameter		Ulcerative colitis	Crohn's disease	*p* value^a^
Has your physician informed you about the connection between IBD and oral lesions? (*n* [%])	Yes	12 (9.0)	64 (33.2)	**< 0.01**
	No	111 (82.8)	116 (60.1)	
	Don't know	11 (8.2)	13 (6.7)	
Do you think your physician is interested in problems with your mouth? (*n* [%])	Yes	28 (20.9)	61 (31.6)	0.09
	No	48 (35.8)	56 (29.0)	
	Don't know	58 (43.3)	76 (39.4)	
Do you think your physician knows how to treat oral lesions? (*n* [%])	Yes	42 (31.3)	70 (36.3)	0.44
	No	36 (26.9)	41 (21.2)	
	Don't know	56 (41.8)	82 (42.5)	
Do you regularly have oral lesions and if so, when was the last time you experienced them? (*n* [%])	No oral lesions	86 (64.2)	100 (51.8)	0.13^b^
	Within the last week	8 (5.9)	18 (9.3)	
	1 to 4 weeks ago	15 (11.2)	22 (11.4)	
	1 to 3 months ago	10 (7.5)	13 (6.7)	
	3 to 6 months ago	3 (2.2)	14 (7.3)	
	≥ 7 months ago	12 (9.0)	26 (13.5)	
Are the oral lesions localized or generalized? (*n* [%])^c^	Localized	31 (64.6)	60 (64.5)	0.99
	Generalized	17 (35.4)	33 (35.5)	
How do the oral lesions occur in relation to your IBD? (*n* [%])^c^	Simultaneously with the flare‐up of the IBD	10 (20.8)	21 (22.6)	0.99^b^
	Before the flare‐up of the IBD	5 (10.4)	11 (11.8)	
	After the flare‐up of the IBD	3 (6.3)	5 (5.4)	
	In no relation to the IBD	30 (62.5)	56 (60.2)	
How painful are the oral lesions? (*n* [%])^c^	Not painful at all	2 (4.2)	4 (4.3)	0.20^b^
	Slightly painful	19 (39.6)	41 (44.1)	
	Average painful	20 (41.6)	24 (25.8)	
	Very painful	7 (14.6)	24 (25.8)	
Are the oral lesions preventing you from eating what you want? (*n* [%])^c^	Always	1 (2.1)	6 (6.4)	0.15^b^
	Very often	0 (0)	9 (9.7)	
	Often	7 (14.6)	13 (14.0)	
	Sometimes	15 (31.2)	28 (30.1)	
	Seldom	17 (35.4)	21 (22.6)	
	Never	8 (16.7)	16 (17.2)	
Do you feel that the oral lesions negatively affect your social activity, sports/leisure, personal relationships? (*n* [%])^c^	Always	1 (2.1)	2 (2.2)	0.47^b^
	Very often	1 (2.1)	4 (4.3)	
	Often	0 (0.0)	7 (7.5)	
	Sometimes	9 (18.7)	16 (17.2)	
	Seldom	14 (29.2)	23 (24.7)	
	Never	23 (47.9)	41 (44.1)	
Do you get any treatment for the oral lesions? (*n* [%])^c^	Yes	2 (4.2)	19 (20.4)	**0.01** b
	No	46 (95.8)	74 (79.6)	
If you get treatment for the oral lesions, who gave it to you? (*n* [%])]^d^^,^ e	Dentist	2 (100.0)	9 (47.4)	0.48^b^
	Gastroenterologist	0 (0.0)	4 (21.1)	1.00^b^
	General practitioner	0 (0.0)	3 (15.8)	1.00^b^
	Myself	0 (0.0)	6 (31.6)	1.00^b^

*Note:* Bold values indicate significance.

Abbreviation: IBD, inflammatory bowel disease.

^a^ Chi‐squared test was applied unless indicated otherwise.

^b^ Fisher's exact test was applied.

^c^ Based on 141 patients with IBD indicating having oral lesions.

^d^ Based on the 21 patients with IBD receiving treatment.

^e^ Multiple answers possible.

### 3.6 PESS

Appendix Tables A1 and A2 present the results from the uni‐ and multivariable binary logistic regression analyses for the primary outcome parameter “PESS ≥ 5,” that is, presence of self‐reported severe periodontitis. In the multivariable analysis, IBD diagnosis/patient group was not significantly associated with the occurrence of self‐reported severe periodontitis (OR 0.733, 95% CI 0.369 to 1.456; *p* = 0.375). The following 3 confounders presented a significant positive association (i.e., higher odds) with the presence of self‐reported severe periodontitis: (1) Fewer teeth (OR 5.172, 95% CI 2.430 to 11.008; *p* < 0.001); (2) higher age (OR 1.136, 95% CI 1.099 to 1.175; *p* < 0.001); and (3) current smoking (OR 5.135, 95% CI 1.810 to 14.574; *p* = 0.002).

### 3.7 Tooth Number

Appendix Tables A3 and A4 present the results from the uni‐ and multivariable binary logistic regression analyses for the primary outcome parameter “tooth number.” In the multivariable analysis, IBD diagnosis/patient group did not have any significant effect on having < 20 teeth (OR 1.327, 95% CI 0.675 to 2.609; *p* = 0.413). The following 2 confounders presented a significant positive association (i.e., higher odds) with having < 20 teeth: (1) Having self‐reported severe periodontitis (OR 5.030, 95% CI 2.470 to 10.245; *p* < 0.001); and (2) a higher age (OR 1.057, 95% CI 1.026 to 1.089; *p* < 0.001); while any kind of education (compared to no or only primary school) was significantly associated with having ≥ 20 teeth (OR ranged from 0.240 to 0.433).

## 4 Discussion

The results of the present questionnaire‐based study indicate a high prevalence of severe periodontitis, tooth loss, and poor OHRQoL in IBD patients in Austria. Specifically, about 34% of the responders reported severe periodontitis, 25% reported < 20 teeth, and 7% reported poor OHRQoL. These results are in accordance with what was observed in similar surveys conducted recently in Denmark and Sweden (Bertl et al. 2022; Bertl et al. 2024b). In the Danish population, based on the responses of about 1100 IBD patients, around 32% reported severe periodontitis, 16% had < 20 teeth, and 6.5% reported poor OHRQL (Bertl et al. 2022), while in the Swedish IBD patients, based on about 800 responses, the corresponding values were around 39%, 19%, and 6.5% (Bertl et al. 2024b).

Data on oral health, including periodontal aspects, from a general Austrian population do not exist; however, the German Oral Health Study (GOHS) is often considered as a valid dataset for comparisons with data collected in Austria. Recently, the sixth edition of the GOHS was published, including detailed data on periodontal diseases and tooth loss (Eickholz et al. 2025; Kocher et al. 2025; Wöstmann et al. 2025). In addition, the data presented in the GOHS on periodontitis are based—amongst others—on the CDC/AAP case definition (Page and Eke 2007), which was also the case definition used to validate the PESS. In the GOHS, severe periodontitis was present among the 35‐ to 44‐year‐old ones in 5.3% and among those 65 to 74 years old in 22.3%. Herein, the overall rate of self‐reported severe periodontitis was about 34%, while it increased to about 75% for those ≥ 55 years old, indicating a strikingly higher prevalence of severe periodontitis. In this context, a high burden of periodontal disease is likely to result in a high tooth loss rate in the long‐term. In the GOHS, about 2% of the 35‐ to 44‐year‐old ones had < 20 teeth and only 0.1% were edentulous, while about 37% of those 65 to 74 years old had < 20 teeth and 5.5% were edentulous. Herein, about 25% of the IBD patients reported having < 20 teeth and 2.1% were edentulous, while for those ≥ 55 years old the corresponding values were 48% and 6.5%, respectively. Thus, despite the fact that age categories in this study and the GOHS are not directly comparable and that differences in disease parameters/determinants may partly reflect differences in oral health care systems (e.g., state insurance coverage/reimbursement), the data support the notion that IBD patients have a relatively higher risk of severe periodontitis and a higher risk of becoming edentulous than the background population. Indeed, in the large‐scale Danish study mentioned above (Bertl et al.), IBD patients reported significantly more periodontal problems, less teeth, and worse oral health, in general, compared to matched, non‐IBD individuals. Furthermore, in a recent ongoing study in Denmark, prevalence of widespread gingival bleeding and deep pockets in newly diagnosed IBD patients was about 2‐ to 3‐times higher, respectively, than in a Danish background population (Bertl et al. 2026).

In general, patients with CD often appear more affected compared to patients with UC (Bertl et al. 2022; Madsen et al. 2023); however, this was less obvious in the current population, for both dental and IBD‐related parameters. Although, patients with CD tended to judge the overall health of their teeth and gums more likely as average or bad, felt more likely uncomfortable about the appearance of their teeth or dentures, and they also spent more money for professional dental care in the past 12 months compared to patients with UC, IBD diagnosis *per se*, that is, CD or UC, did not have any significant effect on the prevalence of self‐reported severe periodontitis and the risk of having < 20 remaining teeth herein (in the regression analysis). This lack of effect when controlling for confounders agrees with our data previously collected in Sweden (Bertl et al. 2024b).

Next to periodontitis and tooth loss, patients with IBD are also affected by oral lesions. The Austrian patients with IBD reported a higher prevalence of problems with oral lesions, that is, almost 50% higher, compared to the Scandinavian populations mentioned above, with about 4 out of 10 patients reporting problems (Table 6). Nevertheless, like in the Scandinavian populations, oral lesions appear in general seldomly addressed by the physicians, with most of the patients reporting not being informed about the occurrence of oral lesions in IBD. UC patients appear more neglected compared to CD patients, with only about 10% reporting having received some information and only about 4% reporting receiving some treatment, while 30% of the CD patients are reporting having received information on oral lesions and about 20% reporting having received some treatment. This is likely due to the fact that CD patients are more often burdened with oral lesions (Katsanos et al. 2015; Sbeit et al. 2020). Interestingly, about 1/3 of the patients receiving some treatment for oral lesions chose the treatment themselves, and only a small fraction of the patients think that their physician is interested in oral lesions (on average about 25%), as it was also the case with the Scandinavian patients. In perspective, in the two latest European Crohn's and Colitis Organization consensus on extra‐intestinal manifestations (Harbord et al. 2016; Gordon et al. 2024), very little information is included about oral health‐related problems, including periodontitis, reflecting the general low knowledge and/or awareness among medical doctors about any association of oral health with general health (Al‐Habashneh et al. 2008; Chatzaki et al. 2023). Altogether, the higher prevalence of severe periodontitis, having < 20 teeth, and oral lesions in this population compared to the Danish and Swedish IBD patients (Table 6), might also explain the finding that the Austrian population had the highest prevalence of poor OHRQoL among these 3 countries; it is well known, that OHRQoL is significantly affected by periodontitis and tooth loss (Craddock 2009; Graziani and Tsakos 2020; Agnese et al. 2025).

**Table 6:** Presentation of the dental and periodontal characteristics of the current (Austrian) and 2 previous cohorts from Denmark (Bertl et al. 2022; Bertl et al. 2024a; Bertl et al. 2023b) and Sweden (Bertl et al. 2024b), based on information collected with similar questionnaires.

Parameter			Austrian IBD cohort (*n* = 327)	Danish IBD cohort	Swedish IBD cohort
Tooth number (*n* [%])	All patients	Edentulous	7 (2.1)	6 (0.6)	8 (1.0)
		1 to 9 teeth	25 (7.7)	14 (1.3)	31 (4.0)
		10 to 19 teeth	47 (14.4)	151 (13.8)	111 (14.1)
		≥ 20 teeth	248 (75.8)	922 (84.3)	636 (80.9)
	≥ 55 years	Edentulous	6 (6.5)	5 (1.4)	7 (1.5)
		1 to 9 teeth	20 (21.7)	9 (2.5)	27 (5.9)
		10 to 19 teeth	18 (19.6)	79 (21.6)	86 (18.6)
		≥ 20 teeth	48 (52.2)	272 (74.5)	342 (74.0)
PESS ≥ 5 (yes; *n* [%])		All patients	110 (33.6)	352 (31.8)	302 (38.5)
		≥ 55 years	69 (75.0)	236 (64.7)	269 (58.2)
OHIP judgment (poor; *n* [%])		All patients	24 (7.3)	72 (6.5)	51 (6.5)
		≥ 55 years	6 (6.5)	19 (5.2)	30 (6.5)
Problems with oral lesions (yes; *n* [%])		All patients	141 (43.1)	323 (29.2)	239 (30.4)
		≥ 55 years	42 (45.7)	93 (25.5)	123 (26.6)

Abbreviations: IBD, inflammatory bowel disease; OHIP, oral health impact profile; PESS, periodontal screening score.

In this context, the present cross‐sectional survey is potentially limited by the fact that the data were collected at a random timepoint after IBD diagnosis, by the lack of a clinical dental examination, and by some risk for selection bias. Nevertheless, regarding the lack of a clinical dental examination, it should be kept in mind that a sensitivity and specificity of about 79% and 75%, respectively, have been reported for PESS (Carra et al. 2018), and that self‐reporting of the number of remaining teeth has been previously proven reliable (Buhlin et al. 2002; Matsui et al. 2016; Similä et al. 2018; Ueno et al. 2018). Furthermore, it might be that patients having oral health problems were more tempted to invest the time in participating voluntarily in such a survey. However, as the questionnaire was disseminated via mail, social media, and an announcement on the homepage of the Austrian Crohn's Disease/Ulcerative Colitis Association (ÖMCCV), it was not possible to calculate an exact response rate; nevertheless, as the ÖMCCV has approximately 1000 members, an approximate response rate of 25%–30% can be assumed, which agrees well with the previous reports (Bertl et al. 2022). In addition, it appears that the responders are rather well representative of the IBD patients in Austria regarding the distribution of several IBD characteristics, such as disease diagnosis, activity, and severity (including IBD‐related surgery) (Gröchenig et al. 2019). Altogether, to support/validate the findings herein and those of the Scandinavian studies mentioned above, longitudinal studies on oral aspects of IBD, as well as interventional studies assessing the possible impact of periodontal treatment on IBD course, are warranted. Nevertheless, this study provides additional evidence supporting the assumption that oral health remains inadequately addressed in patients with IBD, although they are in need of significant dental care. As mentioned above, in the present population, about 3 out of 4 patients with IBD being ≥ 55‐years‐old reported the presence of severe periodontitis, and about 1/4 of all patients reported < 20 teeth. Hence, interdisciplinary care in managing IBD patients is highly warranted. Specifically, a closer collaboration between gastroenterologists and dental professionals might improve the outcome in terms of preventive and therapeutic measures, as shown in other disciplines, such as oral health strategies implemented in primary and cardiovascular care settings (Usmani et al. 2026).

Concluding, and keeping in mind that data are solely self‐reported and collected at a single timepoint, IBD patients in Austria seem to show high prevalence of severe periodontitis, having < 20 teeth, and low OHRQoL, and there is a need for increasing the awareness of medical and dental professionals about oral diseases in IBD patients. Considering the fact that IDB often appears at a relatively young age and that oral diseases are largely preventable, knowledge about the association of IBD with oral health and focus on preventive measures may result in less oral diseases in IBD patients.

## Author Contributions

**Kristina Bertl:** conceptualization, data analysis, interpretation, drafting of the manuscript, organization, funding. **Arghavan Yari:** set‐up and distribution of the questionnaire, data collection, interpretation, critical review of the manuscript. **Anna Riener:** data analysis, interpretation, drafting of the manuscript. **Johan Burisch:** conceptualization, interpretation, critical review of the manuscript, organization. **Nikolaos Pandis:** data analysis, interpretation, critical review of the manuscript. **Hady Haririan:** interpretation, critical review of the manuscript, organization. **Andreas Stavropoulos:** conceptualization, data analysis, interpretation, drafting of the manuscript, organization, funding.

## Ethics Statement

Ethical approval was not required by the local authorities.

## Consent

This is a questionnaire‐based study, and as such participation was de facto voluntary.

## Conflicts of Interest

All authors declare no conflict of interest, but Andreas Stavropoulos is Senior Editor‐in‐Chief and Kristina Bertl is Associate Editor of the journal Clinical and Experimental Dental Research.

## Permission to Reproduce Material From Other Sources

All material was originally created by the authors; thus, no permissions are required.

## Data Availability

All data are available upon reasonable request.

## 1

**Table A1:** Results of the univariable binary logistic regression analyses for the dichotomous outcome parameter “PESS ≥ 5.” An odds ratio (OR) above one indicates higher odds for a PESS ≥ 5 (i.e., presence of self‐reported severe periodontitis); all models have been corrected for age, gender, and smoking status.

Parameter		OR	95% Cl		*p* value
			Lower	Upper	
Patient group	Ulcerative colitis	Ref.			
	Crohn's disease	0.94	0.51	1.74	0.84
Tooth number	≥ 20 teeth	Ref.			
	< 20 teeth	**5.10**	**2.56**	**10.16**	**< 0.01**
Diabetes	Absent	Ref.			
	Present	1.16	0.28	4.80	0.84
Osteoporosis	Absent	Ref.			
	Present	0.53	0.19	1.51	0.24
Rheumatoid arthritis	Absent	Ref.			
	Present	1.97	0.57	6.88	0.29
Ankylosing spondylitis	Absent	Ref.			
	Present	1.25	0.18	8.98	0.82
Psoriasis	Absent	Ref.			
	Present	0.80	0.22	2.97	0.74
Depression	Absent	Ref.			
	Present	1.31	0.59	2.90	0.51
High cholesterol	Absent	Ref.			
	Present	1.03	0.44	2.41	0.95
Cardiovascular disease	Absent	Ref.			
	Present	0.49	0.12	2.02	0.32
Asthma	Absent	Ref.			
	Present	1.31	0.37	4.71	0.68
COPD	Absent	Ref.			
	Present	3.66	0.31	43.33	0.30
Living area	Vienna	Ref.			
	> 100,000 inhabitants	2.01	0.69	5.88	0.20
	40,000 to 100,000 inhabitants	0.22	0.03	1.36	0.10
	10,000 to 40,000 inhabitants	0.68	0.27	1.70	0.41
	< 10,000 inhabitants	0.74	0.36	1.50	0.40
Education	No school/primary school	Ref.			
	High school	0.94	0.47	1.89	0.87
	Higher education up to 3 years	0.69	0.25	1.91	0.47
	Higher education up to 6 years and/or PhD	0.48	0.20	1.19	0.11
Income	**<** 1000 €	Ref.			
	1000 to < 2000 €	1.43	0.57	3.55	0.44
	2000 to < 3000 €	0.45	0.17	1.21	0.11
	≥ 3000 €	1.20	0.35	4.07	0.77
BMI	Score	1.02	0.97	1.08	0.43
Age	Years	**1.14**	**1.11**	**1.17**	**< 0.01**
Gender	Male	Ref.			
	Female	1.13	0.60	2.11	0.70
Smoking	Never	Ref.			
	Former	1.14	0.58	2.21	0.71
	Current	**6.33**	**2.59**	**15.46**	**< 0.01**

*Note:* Bold values indicate significance.

Abbreviations: €, euros; BMI, body mass index; CI, confidence interval; COPD, chronic obstructive pulmonary disease; OR, odds ratio; PESS, periodontal screening score.

**Table A2:** Results of the multivariable binary logistic regression analysis for the dichotomous outcome parameter “PESS ≥ 5.” An odds ratio (OR) above one indicates higher odds for a PESS ≥ 5 (i.e., presence of self‐reported severe periodontitis); the model has been corrected for age, gender, and smoking status.

Parameter		OR	95% Cl		*p* value
			Lower	Upper	
Patient group	Ulcerative colitis	Ref.			
	Crohn's disease	0.73	0.37	1.46	0.38
Tooth number	≥ 20 teeth	Ref.			
	< 20 teeth	**5.17**	**2.43**	**11.01**	**< 0.01**
Living area	Vienna	Ref.			
	> 100,000 inhabitants	1.89	0.58	6.17	0.30
	40,000 to 100,000 inhabitants	0.16	0.02	1.28	0.08
	10,000 to 40,000 inhabitants	0.59	0.21	1.69	0.33
	< 10,000 inhabitants	0.51	0.22	1.19	0.12
Education	No school/Primary school	Ref.			
	High school	1.47	0.66	3.28	0.35
	Higher education up to 3 years	1.04	0.34	3.23	0.94
	Higher education up to 6 years	0.74	0.25	2.13	0.57
Income	**<** 1000 €	Ref.			
	1000 to < 2000 €	1.50	0.53	4.25	0.45
	2000 to < 3000 €	0.43	0.13	1.39	0.16
	≥ 3000 €	1.22	0.30	4.96	0.78
Age	Years	**1.14**	**1.10**	**1.18**	**< 0.01**
Gender	Male	Ref.			
	Female	1.11	0.51	2.43	0.79
Smoking	Never	Ref.			
	Former	1.26	0.59	2.68	0.55
	Current	**5.14**	**1.81**	**14.57**	**< 0.01**
Hosmer–Lemeshow test—*p*‐value: 0.50					

*Note:* Bold values indicate significance.

Abbreviations: €, euros; CI, confidence interval; OR, odds ratio; PESS, periodontal screening score.

**Table A3:** Results of the univariable binary logistic regression analyses for the dichotomous outcome parameter “tooth number.” An odds ratio (OR) above one indicates higher odds to have less than 20 teeth; all models have been corrected for age, gender, and smoking status.

Parameter		OR	95% Cl		*p* value
			Lower	Upper	
Patient group	Ulcerative colitis	Ref.			
	Crohn's disease	1.34	0.72	2.51	0.36
PESS	< 5	Ref.			
	≥ 5	**5.14**	**2.60**	**10.15**	**< 0.01**
Diabetes	Absent	Ref.			
	Present	0.69	0.17	2.82	0.60
Osteoporosis	Absent	Ref.			
	Present	1.40	0.54	3.62	0.49
Rheumatoid arthritis	Absent	Ref.			
	Present	2.30	0.74	7.16	0.15
Ankylosing spondylitis	Absent	Ref.			
	Present	0.41	0.04	3.94	0.44
Psoriasis	Absent	Ref.			
	Present	0.47	0.11	1.93	0.29
Depression	Absent	Ref.			
	Present	1.51	0.71	3.21	0.28
High cholesterol	Absent	Ref.			
	Present	1.20	0.52	2.74	0.67
Cardiovascular disease	Absent	Ref.			
	Present	1.31	0.37	4.68	0.68
Asthma	Absent	Ref.			
	Present	1.75	0.51	5.98	0.37
COPD	Absent	Ref.			
	Present	2.26	0.30	16.97	0.43
Living area	Vienna	Ref.			
	> 100,000 inhabitants	0.99	0.33	3.00	0.99
	40,000 to 100,000 inhabitants	1.06	0.23	4.87	0.94
	10,000 to 40,000 inhabitants	0.78	0.31	1.92	0.58
	< 10,000 inhabitants	0.87	0.44	1.74	0.67
Education	No school/Primary school	Ref.			
	High school	**0.46**	**0.24**	**0.91**	**0.03**
	Higher education up to 3 years	**0.28**	**0.09**	**0.87**	**0.03**
	Higher education up to 6 years and/or PhD	**0.23**	**0.09**	**0.59**	**< 0.01**
Income	**<** 1000 €	Ref.			
	1000 to < 2000 €	1.04	0.42	2.57	0.93
	2000 to < 3000 €	0.65	0.24	1.74	0.39
	≥ 3000 €	1.05	0.31	3.55	0.94
BMI	Score	1.00	0.94	1.06	0.89
Age	Years	**1.10**	**1.07**	**1.13**	**< 0.01**
Gender	Male	Ref.			
	Female	0.79	0.43	1.47	0.46
Smoking	Never	Ref.			
	Former	1.27	0.65	2.49	0.48
	Current	**3.98**	**1.68**	**9.42**	**< 0.01**

*Note:* Bold values indicate significance.

Abbreviations: €, euros; BMI, body mass index; CI, confidence interval; COPD, chronic obstructive pulmonary disease; OR, odds ratio; PESS, periodontal screening score.

**Table A4:** Results of the multivariable binary logistic regression analysis for the dichotomous outcome parameter “tooth number.” An odds ratio (OR) above one indicates higher odds to have less than 20 teeth; the model has been corrected for age, gender, and smoking status.

Parameter		OR	95% Cl		*p* value
			Lower	Upper	
Patient group	Ulcerative colitis	Ref.			
	Crohn's disease	1.33	0.68	2.61	0.41
PESS	< 5	Ref.			
	≥ 5	**5.03**	**2.47**	**10.25**	**< 0.01**
Rheumatoid arthritis	Absent	Ref.			
	Present	2.29	0.69	7.57	0.17
Education	No school/Primary school	Ref.			
	High school	**0.43**	**0.21**	**0.89**	**0.02**
	Higher education up to 3 years	0.32	0.10	1.01	0.05
	Higher education up to 6 years	**0.24**	**0.09**	**0.66**	**< 0.01**
Age	Years	**1.06**	**1.03**	**1.09**	**< 0.01**
Gender	Male	Ref.			
	Female	0.67	0.33	1.35	0.26
Smoking	Never	Ref.			
	Former	1.15	0.55	2.39	0.72
	Current	1.84	0.68	4.95	0.23
Hosmer–Lemeshow test—*p*‐value: 0.59					

*Note:* Bold values indicate significance.

Abbreviations: CI, confidence interval; OR, odds ratio; PESS, periodontal screening score.
